# Comparison of Propofol, Propofol-Remifentanil, and Propofol-Fentanyl Administrations with Each Other Used for the Sedation of Patients to Undergo ERCP

**DOI:** 10.1155/2015/465465

**Published:** 2015-10-21

**Authors:** Candan Haytural, Bahar Aydınlı, Berna Demir, Elif Bozkurt, Erkan Parlak, Selçuk Dişibeyaz, Ahmet Saraç, Ayşegül Özgök, Dilek Kazancı

**Affiliations:** ^1^Anesthesia Clinic, Türkiye Yüksek Ihtisas Education and Research Hospital, Kızılay S. No. 4, Sıhhıye, Altındağ, 06810 Ankara, Turkey; ^2^Anesthesia Clinic, Caycuma State Hospital, Zonguldak, Turkey; ^3^Gastroenterology Clinic, Sakarya University Faculty of Medicine, Sakarya, Turkey; ^4^Gastroenterology Clinic, Türkiye Yüksek İhtisas Hospital, Ankara, Turkey

## Abstract

*Introduction*. Using single anesthetic agent in endoscopic retrograde cholangiopancreatography (ERCP) may lead to inadequate analgesia and sedation. To achieve the adequate analgesia and sedation the single anesthetic agent doses must be increased which causes undesirable side effects. For avoiding high doses of single anesthetic agent nowadays combination with sedative agents is mostly a choice for analgesia and sedation for ERCP. *Aim*. The aim of this study is to investigate the effects of propofol alone, propofol + remifentanil, and propofol + fentanyl combinations on the total dose of propofol to be administered during ERCP and on the pain scores after the process. *Materials and Method*. This randomized study was performed with 90 patients (ASA I-II-III) ranging between 18 and 70 years of age who underwent sedation/analgesia for elective ERCP. The patients were administered only propofol (1.5 mg/kg) in Group Ι, remifentanil (0.05 *μ*g/kg) + propofol (1.5 mg/kg) combination in Group II, and fentanyl (1 *μ*g/kg) + propofol (1.5 mg/kg) combination in Group III. All the patients' sedation levels were assessed with the Ramsey Sedation Scale (RSS). Their recovery was assessed with the Aldrete and Numerical Rating Scale Score (NRS) at 10 min intervals. *Results*. The total doses of propofol administered to the patients in the three groups in this study were as follows: 375 mg in Group I, 150 mg in Group II, and 245 mg in Group III. *Conclusion*. It was observed that, in the patients undergoing ERCP, administration of propofol in combination with an opioid provided effective and reliable sedation, reduced the total dose of propofol, increased the practitioner satisfaction, decreased the pain level, and provided hemodynamic stability compared to the administration of propofol alone.

## 1. Introduction

ERCP (endoscopic retrograde cholangiopancreatography) is an endoscopic process used to visualize the biliary pancreatic ductal system through the injection of a radiopaque contrast medium. Anesthesia support during ERCP is widely accepted and it has become almost a standard practice. Since administering a single-agent during ERCP leads to inadequate sedation and analgesia and thus to excessive drug use and increases in undesirable side effects, using sedative agents in combination has become more widespread [1, 2]. Although there are several studies in the literature reporting that administering propofol in combination with an opioid leads to early awakening from sedation [3, 4], the number of studies on the effects of opioids on the propofol dose is limited [5].

This study was aimed at investigating the effects of administration of propofol alone or in combination with remifentanil or fentanyl on the total dose of propofol during ERCP and on the anxiety level after the process.

## 2. Materials and Methods

The permission for the study was received from the Education Planning Department of Türkiye Yüksek Ihtisas Education and Research Hospital in Ankara, Turkey. The study was performed with 90 patients (ASA I-II-III) who were scheduled to undergo elective ERCP. The participants were between the ages of 18 and 70 years Those younger than 18 and older than 70 years old; pregnant, epileptic, allergic to the medicine to be administered; taking chronic opioids, sedatives, and analgesics; having had a condition requiring emergency intervention; having undergone surgery within the last 72 hours; having psychiatric problems; and/or taking drugs affecting central nervous system (CNS) were excluded from the study.

After peripheral venous access was established in the patients to be treated in the ERCP unit, the patients had intravenous infusion of 0.9% saline and they were followed with noninvasive interventions such as blood pressure (NIBP), electrocardiogram (ECG), blood oxygen saturation, and respiratory rate monitorization. The patients who received O_2_ (4–6 L/min) via nasal oxygen cannula throughout the process were not given any premedication before the process.

The patients were randomly divided into 3 groups of 30 people each. The patients in Group Ι were given only propofol infusion: first loading dose of 1.5 mg/kg then maintenance dose of 1 mg/kg/h. The patients in Group ΙΙ were administered remifentanil infusion of 0.05 *μ*g/kg/min (Ultiva GlaxoSmithKline, Belgium) intravenously 5 min before the process. They were also given propofol infusion: a loading dose of 1.5 mg/kg immediately before the process then maintenance dose of 1 mg/kg/h. The patients in Group ΙΙΙ were administered fentanyl of 1 *μ*g/kg and 1.5 mg/kg loading dose of propofol intravenously 5 minutes before the process. Then they were given 1 mg/kg/h maintenance dose of propofol infusion. The patients in Group I were administered 20 mg of lidocaine intravenously before propofol administration in order to prevent injection pain. In order to maintain Ramsey Sedation Scale (RSS) between 3 and 4, all the patients were given 0.5 mg/kg bolus of propofol when necessary, and the patients in Group III were given 0.01 mg/kg bolus of fentanyl.

Data about all the patients' systolic arterial pressure (SAP), diastolic arterial pressure (DAP), mean arterial pressure (MAP), heart rate, saturation of peripheral oxygen (SpO_2_), RSS, and ECG were recorded at 5 min intervals.

Complications such as SpO_2_ level lower than 95%, hypocapnia, apnea, nausea and vomiting, hypotension, hypertension, and bradycardia observed during the process were recorded, the process was suspended, and the necessary interventions were performed. At the end of the process, the comfort level was graded as very good, good, moderate, and poor by the gastroenterologist.

When the RSS level was 2 after the process, the patient was taken to the recovery room. In the recovery room, Aldrete and NRS (numerical rating scale score) were assessed at 10 min intervals and the total length of stay in the recovery unit was recorded. When the Aldrete score was 9 points, the patient was transferred to the ward from the recovery room.

Statistical analyses were performed with SPSS 11.5. The significance of the difference between the group means was assessed with the One-Way ANOVA. The significance of the difference between the means was assessed with the Kruskal-Wallis test. Categorical variables were assessed with Pearson's chi-square test or Fisher's exact test.

Hemodynamic parameters were assessed by repeated measures analysis of variance. Whether the effects of medication on the changes in hemodynamic measurements in terms of follow-up (monitorization) times varied statistically significantly was assessed using the Greenhouse-Geisser test statistics. *p* value of <0.05 was considered statistically significant.

## 3. Results

The present study included 90 patients (ASA I-II-III) who underwent ERCP. The patients were divided into three groups of 30 patients each. Two patients in Group 1 were excluded from the study because of the drop in their saturation levels during the process.

No statistically significant differences were determined between the groups in terms of demographic characteristics such as age, body weight, and gender (*p* = 0.885/*p* = 0.391/*p* = 0.113) (Table 1).

No statistically significant differences were determined between the groups in terms of diagnosis, ASA, additional disease, and process time (Table 1).

No statistically significant differences were detected between the groups in terms of changes in SAP, DAP, MAP, and saturation levels throughout the follow-up (monitorization) (*p* > 0.05). The differences between the groups in terms of the changes in Ramsey scores were not statistically significant either (*p* > 0.0033) (Table 2).

In terms of total propofol doses administered during the follow-up (monitorization), the difference between Group I and Group II and between Group I and Group III and Groups II and III was statistically significant (*p* < 0.001) (*p* < 0.003). Also there was statistically significant difference between the groups during the follow-up (monitorization) period in terms of pain levels (*p* < 0.05). The patients in Group I suffered from the pain most, whereas the patients in Group II had the least pain (Table 3).

**Figure 1:**
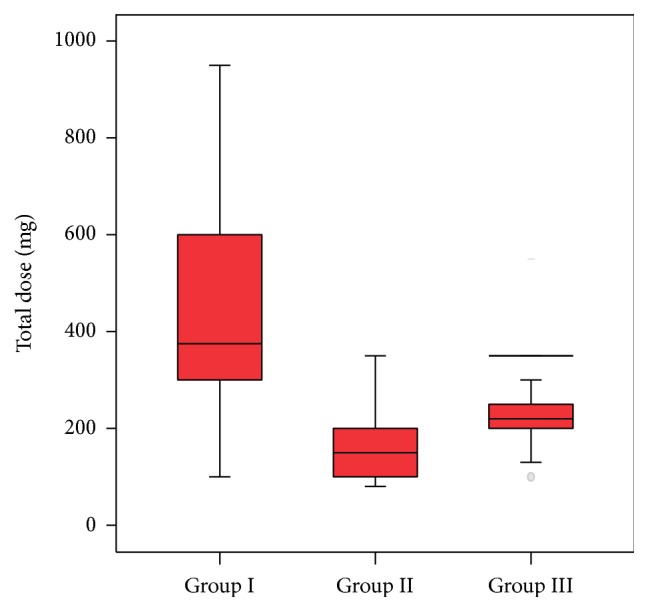
The total dose of propofol used during the follow-up period according to the groups.

The comparison of the groups in terms of the total dose of propofol revealed that it was 375 mg in Group I, 150 mg in Group II, and 245 mg in Group III (Figure 1).

**Figure 2:**
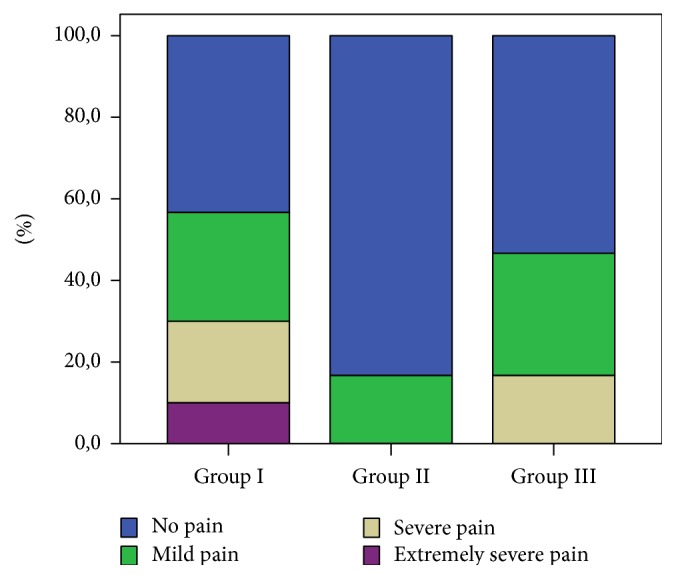
Pain levels during the follow-up period according to the groups.

Comparison of severe pain levels during the follow-up (monitorization) period indicated a statistically significant difference between Group I and Group II (*p* < 0.05). Extremely severe pain was detected only in Group I. In Group II, neither severe nor extremely severe pain was detected. In Group III, extremely severe pain was not detected (Figure 2).

## 4. Discussion

The total doses of propofol administered to the patients in this study were as follows: 375 mg in Group I, 150 mg in Group II, and 245 mg in Group III. The group in which the highest dose of propofol was administered was Group I to which propofol was administered alone. In Lee et al.'s study in which the patients underwent ERCP, the patients in the first group were administered only propofol whereas the patients in the other group were administered midazolam, fentanyl, and/or meperidine in addition to propofol. The total dose of propofol administered was significantly higher in the first group which was administered only propofol than that in the other group to which propofol was administered in combination with other agents [5].

There was a significant difference between the pain levels of Group I and Group II. In Group I, while 13 patients had no pain, the pain level was mild in 8 patients, severe in 6 patients, and extremely severe in 3 patients. In Group II, to which remifentanil and propofol were administered, there was no pain in 25 patients, but 5 patients had mild pain. No patients reported severe or extremely severe pain. In Group III, to which propofol and fentanyl were administered, 16 patients had no pain, 9 patients had mild pain, and 5 patients had severe pain. No patients in Group III reported extremely severe pain. In the literature, there are studies indicating that the patients administered propofol alone suffered more severe pain compared to the patients administered propofol in combination with an opioid [6]. In our study too, the patients in the propofol only group suffered pain most.

Since there could be a significant decrease in oxygen saturation in patients receiving anesthesia support during endoscopy procedures, 4 to 5 liters of nasal oxygen was administered to each patient as indicated in the literature [5, 7]. In our study, two patients in Group I were excluded from the study because they experienced a drop in SpO_2_ although they were provided with nasal oxygen support.

Under conscious sedation, patients are able to maintain protective airway reflexes and can recover quickly. Rapid recovery is an advantage not only for the patient but also for hospitals and day surgery units where rapid patient circulation is desired. Conscious sedation lays the grounds for some interventions and ensures the patient's collaboration with the physician; therefore, it is more advantageous than general anesthesia is. Reducing anxiety and creating amnesia make the patient feel more relaxed and thus ensure favorable conditions necessary for the intervention. Medication used in conscious sedation should have minimum side effects, should depress the patient's consciousness level in a controlled manner, should prevent airway reflexes from being suppressed, should not cause respiration suppression, should ensure early and high quality recovery after the process, should have inactive metabolites, and should not necessitate resedation [8, 9].

In our study too, through the administration of propofol and opioids in given doses, adequate depth of anesthesia was obtained, the comfort necessary for the process was ensured, and no problem was encountered regarding patient recovery. In our study, the modified Aldrete recovery scoring was used and no significant differences were determined between the groups. In one study, the researchers compared sevoflurane and propofol in patients who had outpatient surgery under anesthesia and reported no differences between the groups regarding the patients' early recovery and cognitive functions (remembering and telling their names, ages, dates of birth, etc.) [10].

When the side effect profiles were compared, no side effects were observed in any of the three groups. In studies conducted with propofol, the most common side effect is propofol injection pain. The incidence of pain on injection of propofol ranges between 30% and 70% in case lidocaine or fentanyl is not administered [11]. In our study, in order to prevent pain on injection of propofol, the patients in Group I were administered 20 mg of lidocaine intravenously prior to injection of propofol, and thus the patients suffered no pain. Administration of opioids in the other two groups before the injection of propofol may have prevented the formation of injection pain [12].

Another side effect seen in patients receiving sedation is nausea and vomiting. However, in our study, neither nausea nor vomiting was observed in any patient. Patients' not experiencing nausea and vomiting may have been due to the antiemetic properties of propofol. Amornyotin et al. used propofol as a sedation agent during ERCP and observed neither nausea nor vomiting. They also attributed this result to the antiemetic properties of propofol [13, 14].

The depth of sedation was at such a level as to maintain Ramsey Sedation Scale (RSS) between 3 and 4 which was in all patients during the process. Comparison of Ramsey scores obtained during follow-up (monitorization) indicated no significant differences between the groups. In our literature review, we could not find any other study using Ramsey Sedation scoring.

During ERCP, stimulation, discomfort, and pain levels may vary. Achieving an optimum level of sedation may also be hindered by patient-specific sensitivity. ERCP procedure usually takes longer and is technically more challenging than other gastrointestinal endoscopy procedures; therefore, it requires deep sedation level [15]. Depending on their own preferences and the type of anesthesia monitorization, clinicians may administer boluses* at different doses*.

In our study, there were no statistically significant differences between the groups in terms of the satisfaction of the gastroenterologist who performed the process. However, the gastroenterologist's satisfaction was higher in Group II than in Group I and Group III. The fact that all the interventions were performed by the same gastroenterologist who did not know what agent was administered and that the assessments were made by the same person eliminated the possibility of person-related differences. In their study of 61 patients who underwent ERCP, Mazanikov et al. administered propofol, remifentanil, and alfentanil and observed no differences between the groups in terms of patient and endoscopist satisfaction [16].

The most significant cardiovascular effect of propofol during the induction of anesthesia is a drop in the arterial blood pressure. In our study, differences between the groups were not statistically significant either although there was a decrease in MAP, DAP, and SAP values in all the three groups after the administration of the loading dose of propofol. In their study, Gazdag et al. administered etomidate and propofol to the patients during electroconvulsive therapy and reported that MAP values decreased significantly with propofol administration [17]. In their study, Falk and Zed administered etomidate, propofol, thiopental, etomidate, and midazolam for sedation during cardioversion procedures and determined significant decreases in blood pressure levels with all the medicines except for etomidate [18].

In our study, we considered remifentanil as the most appropriate opioid agent because it led to maximum reduction in the pain level and in the amount of propofol to be administered and its side effects were not different from those of the others. Ince et al. divided hematooncological pediatric patients into two groups, administered remifentanil  +  propofol combination to the first group and propofol  +  fentanyl combination to the second group for sedation, and determined better sedation in the first group during early awakening [3]. Kramer et al. divided oral and dental surgery patients into two groups, administered propofol  +  ketamine combination to the first group and propofol  +  remifentanil combination to the second group for sedation, and determined more effective results in the second group [4].

In conclusion, while providing adequate sedation during ERCP, practitioners should avoid administering sedatives excessively and try to minimize the side effects associated with the administration of excessive sedatives. In line with the findings of our study, it can be said that administration of propofol in combination with an opioid rather than as a single agent to ERCP patients ensured effective and reliable sedation, reduced total dose of propofol, increased practitioner satisfaction, decreased the pain level, and ensured hemodynamic stabilization. We consider remifentanil as the most appropriate opioid agent because it reduces the pain level and the amount of propofol to be administered to the greatest extent and is not different from other agents in terms of side effects.

## Figures and Tables

**Table 1:** Demographic and clinical features of the patients according to the groups.

Variables	Group I	Group II	Group III	*p* value
Age	51.6 ± 12.9	52.1 ± 16.4	50.3 ± 13.3	0.885
Gender M/F	18/12	18/12	11/19	0.113
Weight	73.8 ± 16.7	72.9 ± 11.8	69.1 ± 12.7	0.391
ASA 1/2/3	14/12/4	10/16/4	10/20/0	0.649
Diagnosis				0.195
Liver pathology	16 (%53.3)	19 (%63.3)	23 (%76.7)	
Pancreas pathology	4 (%13.3)	6 (%20.0)	4 (%13.3)	
Others	10 (%33.3)	5 (%16.7)	3 (%10.0)	
Additional disease	14 (%46.7)	19 (%63.3)	18 (%60.0)	0.387
Process time	25 (15–50)	25 (15–50)	25 (15–50)	0.595

**Table 2:** Changes in Ramsey scores in 5th, 10th, 15th, 20th, and 25th minutes according to the groups.

Follow-up time	Group I	Group II	Group III	*p* value^a^
5 minutes	2 (1–3)	1 (1-2)	1.5 (1-2)	0.242
10 minutes	3 (1–3)	2 (1–3)	2.5 (2-3)	0.539
15 minutes	3 (1–3)	2 (1–3)	2.5 (2-3)	0.329
20 minutes	3 (1–3)	2 (1–3)	2 (1–3)	0.110
25 minutes	2 (1–3)	1 (0–3)	1 (0–3)	0.012

According to a = Bonferroni corrections results for *p* < 0.0033 accepted as statistically significant.

**Table 3:** Distribution of groups in terms of other clinical results.

Follow-up time	Group I	Group II	Group III	*p* value^a^
Total dose of propofol (mg)	375 (100–950)^a,b^	150 (80–350)^a,c^	245 (100–550)^b,c^	<0.05
Aldrete score	9 (9-10)	9 (9-10)	9 (9-10)	0.104
Pain				0.002
No pain	13 (%43.3)^a^	25 (%83.3)^a,c^	16 (%53.3)^c^	
Mild	8 (%26.7)	5 (%16.7)	9 (%30.0)	
Severe	6 (%20.0)^a^	0 (%0)^a^	5 (%16.7)	
Extremely severe	3 (%10.0)	0 (%0)	0 (%0)	
Quality of surgery				
Well	8 (%26.7)	3 (%10.0)	6 (%20.0)	
Very well	22 (%73.3)	27 (%90.0)	24 (%80.0)	

a = difference between Group I and Group II was statistically significant (*p* < 0.05), b = difference between Group I and Group III was statistically significant (*p* < 0.001), c = difference between Group II and Group III was statistically significant (*p* < 0.05).
